# Bioinformatic pipeline to identify candidate mRNA transcripts targeted by the putative Regulated Ire1-Dependent Decay pathway of *Dictyostelium discoideum* .

**DOI:** 10.17912/micropub.biology.002098

**Published:** 2026-06-23

**Authors:** Connor Bingham, Elias Taylor-Cornejo

**Affiliations:** 1 Department of Biology, Randolph–Macon College, Ashland, VA, United States

## Abstract

Inositol-requiring protein 1 (Ire1) is a eukaryotic stress sensor that counteracts the buildup of unfolded proteins in the endoplasmic reticulum (ER) by activating the Unfolded Protein Response (UPR) via a specific ribonuclease (RNase) activity. The amoeba *Dictyostelium discoideum* relies on an *ire1* ortholog, *ireA* , to survive ER stress, but the mRNA transcripts targeted by the IreA ribonuclease remain unknown. In this work, we developed a bioinformatic pipeline that identified 21 mRNA transcripts of *D. discoideum* that contain a consensus Ire1 cut site found within a secondary mRNA hairpin loop structure and have the potential to be cut by IreA.

**Figure 1. Predicted stem-loop structures containing Ire1 cleavage sites in candidate RIDD transcripts:** Predicted secondary structures of mRNA hairpin loops containing the consensus Ire1 cleavage site for the 21 candidate RIDD transcripts identified in *D. discoideum* . Two known Ire1 cleavage targets from *S. pombe* are included as representative validation examples: *ema19* , for which the pipeline identified an exact match to the known cleavage site, and *hrf1* , for which the pipeline identified the expected TGC cut site on a predicted stem-loop structure. All sequences are represented as cDNA; the mRNA cleavage sequence UGC therefore appears as TGC. Genes with multiple predicted cleavage sites are shown as separate diagrams labeled site 1, site 2, or site 3. For clarity, gene identifiers beginning with DDB_G0 have been shortened to G0 followed by the unique identifying number. Red nucleotides indicate the predicted TGC cleavage sequence with the red line indicating the cleavage site. RNA secondary structures were predicted by RNAfold and visualized with the software RNA 2D Template (R2DT) (McCann et al. 2025).

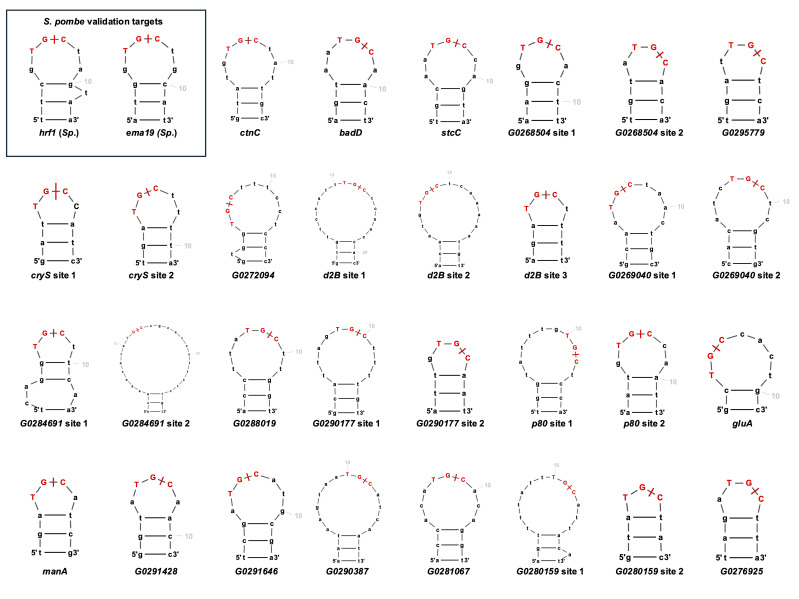



## Description

Within eukaryotic cells, transmembrane and secreted proteins are made by ribosomes located on the endoplasmic reticulum (ER) (Alberts et al. 2002). For these critical proteins to be functional, they must be properly folded by chaperones located within the ER (Alberts et al. 2002). However, when an ER attempts to make too many proteins at once, it causes ER stress by overwhelming the protein synthesis machinery, which can be lethal to cells if not mitigated (Walter and Ron 2011). The Unfolded Protein Response (UPR) is the system responsible for resolving ER stress by sensing and counteracting the buildup of unfolded proteins in the ER (Walter and Ron 2011).

Inositol-requiring enzyme-1 (Ire1) is a highly conserved UPR sensor that is vital for surviving ER stress (Cox and Walter 1996). Ire1 is a ribonuclease (RNase) that is capable of detecting the buildup of unfolded proteins in the ER and responds by cutting specific ER-targeted mRNAs to trigger mechanisms that mitigate ER stress (Cox and Walter 1996; Oikawa et al. 2010; Maurel et al. 2014). The mRNA transcripts that are cut by Ire1 all contain a specific Ire1 cut sequence (UGC) present on an mRNA hairpin loop structure (Kimmig et al. 2012). The mRNA transcripts cut by Ire1 also typically possess a signal peptide sequence that directs them to the ER via the secretory pathway and brings them in contact with Ire1, which resides in the ER membrane (Coelho and Domingos 2014). There are currently two primary mechanisms by which Ire1 cuts mRNAs in response to ER stress in yeast: mRNA splicing and regulated Ire1-dependent mRNA decay (RIDD) (Cox and Walter 1996; Maurel et al. 2014). In mRNA splicing, Ire1 removes an intron from a specific mRNA transcript, *hac1* , which enables the translation of the Hac1 transcription factor that upregulates genes associated with improved protein folding (Cox and Walter 1996). For splicing of *hac1* to occur, Ire1 cuts *hac1* mRNA transcripts in two separate hairpin loops that are then joined by RNA ligases (Sidrauski et al. 1996). On the other hand, in RIDD, Ire1 assists to cut and destroy ER-targeted mRNA transcripts to reduce unnecessary protein production at the ER until proper protein folding can be restored (Maurel et al. 2014). In this method, Ire1 cuts these mRNA targets at a single hairpin loop, where they are subsequently degraded by exonucleases (Hollien and Weissman 2006; Maurel et al. 2014).

In metazoans, such as fruit flies and mammals, Ire1 can use both mRNA slicing and RIDD to regulate the UPR in response to ER stress (Moore and Hollien 2015). However, in microbes such as yeast, Ire1 appears to mainly use either mRNA splicing or RIDD to activate their UPR. For example, the yeast species *Saccharomyces cerevisiae* exclusively performs *hac1* mRNA splicing, not RIDD, in response to ER stress (Niwa et al. 2004). However, a closely related yeast species *Schizosaccharomyces pombe* lacks an ortholog for *hac1* and therefore cannot perform *hac1* mRNA splicing; instead *, S. pombe* responds to ER stress primarily through the RIDD pathway (Kimmig et al. 2012).

Previous studies have shown that the amoeba *D. discoideum* can activate the UPR via an *ire1* ortholog called *ireA* , which is required to survive ER stress, but the specific RNase function (mRNA splicing or RIDD) is unknown (Domínguez-Martín et al. 2018). Furthermore, the mRNA transcripts that are cut by IreA in response to ER stress have not been identified, and *D. discoideum* lack an ortholog of *hac1* (Domínguez-Martín et al. 2018). Due to the lack of a *hac1* ortholog, we hypothesize that IreA responds to ER stress using the RIDD mechanism. To test this hypothesis, we developed a bioinformatic pipeline to identify ER-targeted mRNA transcripts from *D. discoideum* that contain an Ire1 cut site (UGC) found on a predicted mRNA hairpin loop structure that have been shown to be downregulated in an IreA-dependent manner in response to ER stress.

We first evaluated the effectiveness of our bioinformatic pipeline by using it to identify Ire1 cut sites present in mRNA hairpin loops of transcripts from *S. pombe* that are known to be cleaved by Ire1 *.* Like *D. discoideum* , *S. pombe* also lacks a *hac1* orthologue, and is the best microbial reference organism that responds to ER stress using RIDD (Kimmig et al. 2012). Our bioinformatic pipeline was validated against the full set of 11 known mRNA transcripts cleaved by Ire1 in *S. pombe* and correctly identified 8 of the 11 known targets (Kimmig et al. 2012; Guydosh et al. 2017; Li et al. 2018) (Extended Data Table 1). While identification of all 11 targets would be ideal, we considered this level of accuracy sufficient to generate novel biological insights when applied to *D. discoideum* , as the pipeline demonstrated a clear ability to identify the structural and sequence features characteristic of RIDD transcripts.

We then used the validated bioinformatic pipeline to search the *D. discoideum* cDNA library for transcripts that contain an Ire1 consensus cut sequence (UGC) present on a predicted mRNA hairpin loop structure. Our bioinformatic pipeline identified 4128 out of the 13,266 *D. discoideum* transcribed genes having a “UGC” sequence located on an mRNA hairpin-loop. We narrowed down the list of 4128 potential RIDD transcripts to 41 transcripts by cross-referencing a published RNA-seq dataset reporting 96 unique transcripts that are downregulated in an IreA-dependent manner during ER stress in *D. discoideum* (Domínguez-Martín et al. 2018). Of the 41 potential RIDD transcripts, 21 of them had a greater than 95% probability of possessing a standard Sec/SPI signal peptide sequence, indicating that they were extremely likely to be targeted to the ER (Extended Data Table 2). The location of the UGC cleavage site within the predicted stem-loop structure was confirmed for each of the 21 candidate RIDD transcripts using the RNA 2D Templates (R2DT) software (McCann et al. 2025) (Figure 1). There does not appear to be a specific nucleotide position of the UGC cut sequence within the hairpin loop, or size of the hairpin loop structure, which is consistent with previously characterized RIDD targets from *S. pombe* (Teufel et al. 2022).

Currently, these 21 candidates have not been studied extensively, and their functions remain largely unknown (Extended Data Table 2). Thirteen of the candidates are categorized as proteins of completely unknown function. DDB_G0269040 is broadly associated with reproduction (Eichinger et al. 2005) and DDB_G0281067 is broadly associated with phagocytosis (Gotthardt et al. 2006). DDB_G0288019 is associated with tRNA inosine editing, which allows for the efficient translation of certain codons (Paris et al. 2012). D2B is associated with chemical sensing and breakdown (Baumgardner et al. 2018). Three candidates are associated with responses to bacteria, including p80 through copper transport (Hao et al. 2016), GluA through beta-glucosidase activation (Dimond and Loomis 1976; Munoz-Ruiz et al. 2024), and ManA through alpha-mannosidase activation (Free and Schimke 1976; Munoz-Ruiz et al. 2024). One potential mRNA target, *cryS* , is associated with a significant number of functions within *D. discoideum* , including fruiting body formation and endocytosis (Yuan and Chia 2000). Aside from being synthesized at the ER, the reason for cleaving these 21 specific RIDD transcripts is not entirely clear but could reveal novel insights into how *D. discoideum* responds to ER stress.

Interestingly, 55 of the 96 IreA-dependently downregulated transcripts previously identified (Domínguez-Martín et al. 2018) were not identified as containing a consensus UGC cleavage site within a predicted hairpin loop. We propose two possible explanations for these unaccounted transcripts. First, their downregulation may be an indirect consequence of IreA cleaving a master positive regulator, resulting in reduced expression of its downstream gene targets. Alternatively, IreA may splice an uncharacterized mRNA in *D. discoideum* , producing a transcription factor that indirectly downregulates UPR target genes, analogous to *hac1* mRNA splicing in *S. cerevisiae* . Distinguishing between these possibilities represents an important direction for future experimental work.

A third explanation for the unaccounted transcripts is an important limitation of this bioinformatic pipeline: RNA secondary structure prediction determined purely from nucleotide sequence is not always accurate, which likely accounts for both some of the 55 unaccounted transcripts and the three *S. pombe* targets not recovered during validation. Incorporating experimental genome probing data, such as Selective 2'-Hydroxyl Acylation analyzed by Primer Extension (SHAPE) reactivity data, as structural constraints into RNAfold would likely improve prediction accuracy (Lorenz et al. 2011; Sloma and Mathews 2015). Performing such probing experiments in *D. discoideum* represents an important direction for future work to validate and refine the candidate RIDD targets identified here. Nonetheless, this bioinformatic tool can be broadly applicable to search for a specific nucleotide sequence within a specific RNA structural context.

## Methods

**Bioinformatic pipeline to identify mRNA substrates of IreA**

We designed a bioinformatic pipeline to identify mRNA transcripts from *D. discoideum* with the following criteria: (1) must contain at least one instance of the RIDD consensus cut sequence “UGC” (2) the cut sequence must be found within a mRNA hairpin loop structure (3) the mRNA must be downregulated in an IreA-dependent manner during ER stress (4) the mRNA was preferentially selected if it possessed a signal peptide sequence targeting it to the ER via the secretory pathway, as ER-targeted transcripts are most likely to come into contact with IreA at the ER membrane.

A cDNA library representing all known *D. discoideum* mRNA transcript sequences was obtained from GenBank (Eichinger et al. 2005). We created a Python program, named “CutSequenceFinder” that can search cDNA libraries to identify specified sequences that are predicted to be present within RNA hairpin loop structures (available at: https://github.com/Connor-Bingham/CutSequenceFinder/releases/tag/v1.0 ). To determine secondary structures, stem loops were predicted using RNAfold (ViennaRNA Package v2.0, Lorenz et al. 2011) with the Turner 2004 nearest-neighbor thermodynamic parameters (Mathews et al. 2004), applied at 37°C and a salt concentration of 1.021 M NaCl. Stem loop structures were defined as hairpins containing a minimum of 3 unpaired nucleotides in the loop, consistent with the Turner 2004 nearest-neighbor rules. All stem loops present in the minimum free energy (MFE)-predicted secondary structure were retained; no additional energetic cutoff was applied. The standard output of RNAfold was redirected to a text file to facilitate continued processing. After using CutSequenceFinder to identify transcripts that contain the RIDD cut sequence “UGC” on an RNA hairpin loop, we selected the *D. discoideum* transcripts previously shown to be downregulated during ER stress in an IreA-dependent manner by cross-referencing a published RNA-seq dataset (Domínguez-Martín et al. 2018). Finally, we selected the transcripts that are most likely being targeted to the ER secretory pathway using the SignalP 6.0 webservice to identify the remaining mRNA’s with a predicted N-terminal signal peptide sequence (Teufel et al. 2022). Candidate RIDD transcripts were then visualized with RNA 2D Templates (R2DT) software to confirm the predicted location of the UGC cut sequence within the mRNA hairpin loop (McCann et al. 2025).

## Data Availability

Description: Extended Data Table 1. Ire1 cleavage sites of known S. pombe transcripts.. Resource Type: Dataset. DOI: https://doi.org/10.22002/61ery-d4b13 Description: Extended Data Table 2. Potential cleavage sites of D. discoideum transcripts.. Resource Type: Dataset. DOI: https://doi.org/10.22002/w0vme-dy280
